# Experimental and Numerical Investigation of Deformable Concrete Median Barrier

**DOI:** 10.3390/ma12193176

**Published:** 2019-09-27

**Authors:** Jaeha Lee, Yoseok Jeong, Kyeongjin Kim, Ilkeun Lee, WooSeok Kim

**Affiliations:** 1 Department of Civil Engineering, National Korea Maritime and Ocean University, 727 Taejong-ro, Youngdo-gu, Busan 49112, Korea; jaeha@kmou.ac.kr; 2 Research Institute for Construction Disaster Prevention, Chungnam National University, 99 Daehak-ro, Yuseong-gu, Daejeon 34134, Korea; yosoksi@gmail.com; 3 Department of Civil and Environmental Engineering, National Korea Maritime and Ocean University, 727 Taejong-ro, Youngdo-gu, Busan 49112, Korea; kkj4159@naver.com; 4 Structures Research Group, Korea Expressway Corporation Research Institute, 922 Dongbudae-ro, Hwaseong, Gyeonggi-do 20896, Korea; ilk@ex.co.kr; 5 Department of Civil Engineering, Chungnam National University, 99 Daehak-ro, Yuseong-gu, Daejeon 34134, Korea

**Keywords:** deformable, barrier, full-scale test, shock absorber, collision model

## Abstract

In South Korea, the number of vehicles is gradually increasing. The number of heavy vehicles in 2010 increased up to 19% in less than five years. Therefore, the chances of heavy vehicle-concrete median barrier (CMB) collision also became higher than in the past; therefore, a need to study a stricter design level for improving the current CMB (CMB-15) under harsher environments arose. Accordingly, in the present study, a new concrete median barrier was designed under a stricter impact severity, SB6(420 kJ), compared to the current design impact severity, SB5-B (270 kJ). In particular, shock absorbing devices to absorb impact energy were applied to the CMB. An empty space allows the dowel bars to deform and absorb collision energy. Therefore, deformable CMB was designed and tested. The key parameters selected in our study were dowel bar and wire-mesh. A series of numerical analyses were conducted to evaluate the proposed new deformable CMB designs with shock absorbers. Finally, the optimal design, CMB-17S, was proposed after several evaluations of the proposed designs and a full-scale field test. It was found that, although the developed model did not accurately predict the impact sequence due to certain differences between the actual truck and the truck model, the permanent deformation after collision could be well predicted. Based on the observations from a full-scale impact test, it was recommended that the top part of the CMB should be strengthened since major volume loss occurred due to local impact, which appeared to be due to punching shear failure.

## 1. Introduction

In South Korea, the number of vehicles that use the highway is gradually increasing, and the rate of increase is also growing according to Kim et al. [1]. In another study, Kim et al. [2] mentioned that the number of heavy vehicles in 2010 increased by 19% in less than five years. The chances of vehicle-concrete median barrier (CMB) collision in South Korea seem to be higher than those in the past and, accordingly, a new CMB design under a stricter design level was developed in the current study.

**Figure 1:**
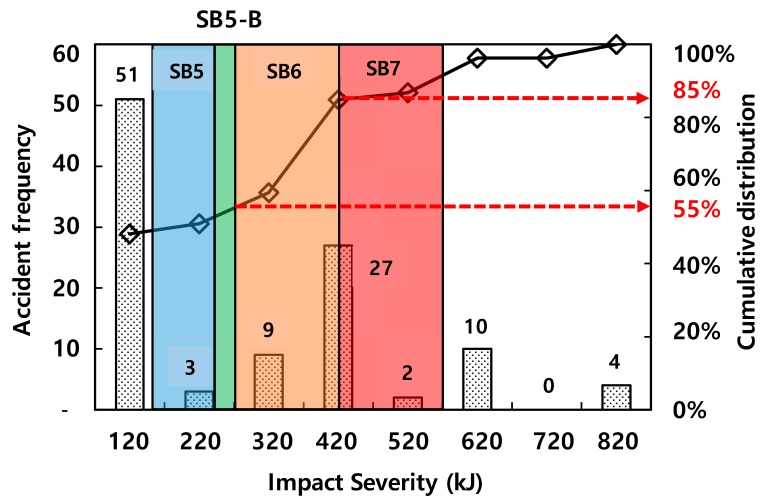
Accident frequencies depending on various impact severities [3].

Figure 1 shows the number of vehicle-CMB collision accidents depending on various levels of impact severities used in South Korea [3]. Among those, the SB5-B (270 kJ) has the highest impact severity, which is currently used in South Korea for the CMB in the expressway. This indicates that the current CMB (CMB-15) can cover only 55% of the total accidents. However, the impact severity, SB6, which is equivalent to 420 kJ, can cover 85% of the accidents based on data. Accordingly, in the present study, a high performance CMB (CMB-17S) under harsh environments such as SB6 was designed in order to reflect the need for improvement of the current CMB (CMB-15) in South Korea.

## 2. Development of High-Performance Concrete Median Barrier (CMB-17S)

In order to develop a high-performance concrete median barrier (CMB-17S), a numerical model was developed first using LS-DYNA, and a key parametric study was conducted. The name (CMB-17S) of the high-performance concrete median barrier denotes that the developed CMB was tested at full scale in the summer of 2017.

The simulation results were compared with the data from the previously conducted field tests, namely, CMB-15 (designed for SB5-B) and CMB-16 (designed for SB5-B (20A)), Kim et al. [1] for verification. Next, the combination of key parameters that showed the best estimates for volume loss and lateral displacement of the two CMBs, namely, CMB-15 and CMB-16, was selected.

Volume loss is defined as the volume of broken pieces of concrete that fall apart from the original concrete body. Therefore, less volume loss means less chance of secondary accident by flying or broken concrete fragmentations. The volume loss (%) ratio was estimated using Equation (1). All the proposed CMBs in our study were evaluated with respect to volume loss.

(1) $$Volume\;loss\left(\%\right)=\frac{Volume\;of\;deleted\;concrete\;elements}{CMB\;volume\;for\;1.0\;m\;length}\times 100$$

After verification of the developed model, section design of CMB-17S was conducted using the model. The size of wire-mesh and the size of the dowel bar in shock absorbers were selected as the key design parameters. The shape of the CMB was not changed, since it was originally developed for the occupant protection performance. The spacing between the dowel bars was also not changed, as the optimal design of the spacing was already found by Kim et al. [4].

### 2.1. Model Development and Verification

In order to develop CMB-17S, first, verification of the model was conducted in combination with certain key parameters. Based on Kim et al. [2,5], the selected key parameters were Erode, B, fracture energy of concrete, repow, and overstress, which are also the input parameters of the concrete material model, CSCM, developed by Murray [6,7]. Other models, such as the K&C model, the Winfrith model [8], and the Karagozian and Case Concrete Damage Model Release 3 [9], can be also considered as material models of concrete. However, the CSCM has more advantages than the other models stated in this study, since it has capability of strain rate, non-linear behavior in both tensile strain and compression, better control of tensile strain softening, and, best of all, it was originally developed for road side safety on highways by the Federal Highway Administration (FHWA). The analysis results were compared with the full scale collision test on the previously developed CMBs, which was developed based on SB5-B (CMB-15) and SB5-B(20A) (CMB-16) introduced by Kim et al. [2]. For estimation of concrete fracture energy, equations from CEB-FIP [10] and FIB model codes [11] were used. Furthermore, the fracture energy of the concrete was directly measured from the three-point bending test based on America Concrete Institute committee 446 report 5 (ACI 446-5) [12] and Lee and Lopez [13]. The experimentally obtained values of the fracture energies for CMB-15 and CMB-16 were 148 N/m and 155 N/m, and those values were used for input values of the analysis. These analytical results were compared with the calculated fracture energy values based on CEB-FIP [10] and FIB model codes [11], respectively. The design compressive strengths of concrete for CMB-15 and CMB-16 were 27 MPa and 30 MPa, respectively. These values were used for estimation of the fracture energies using CEB-FIP [10] and FIB [11] equations. The experimentally obtained value of the concrete compressive strength of CMB-16 was 38 MPa.

Another important key parameter is overstress. The overstress limits the dynamic increment factor at high strain rate in compression and tension separately. In the current study, the range of stress value was considered from 30 to 40 MPa. A more detailed discussion and the specific equations on overstress can be found in Murray [6,7]. The other key parameters, namely, Erode, B, and repow, are found in Lee et al. [14] and Kim et al. [2]. Based on these five selected parameters, a total of 76 cases were analyzed. Among these 76 cases, 38 analysis results from CMB-15 models were compared with the field test results of CMB-15. The other 38 analysis results from CMB-16 models were compared with the field test results of CMB-16 [4]. The obtained results from the analyses were total loss of volume and lateral displacement of deformable CMB caused by the installation of dowel bar type shock absorbers [2]. A combination of the selected five key parameters showed the smallest error in both volume loss and lateral displacement. The selected model comprised Erode 1.1, B 10, fracture energy 155 N/m, repow 3, and overstress 30 MPa. It was found that the selected model accurately predicted the CMB-16. However, for the CMB-15, the obtained results were always larger than the actual experimental values, which is acceptable for the conservative design approach. Since, CMB-17S in the current work was designed following SB6(420 kJ) impact severity, which is more relevant to SB5-B(20A) (456 kJ) than SB5-B (270 kJ), the selected parameters for CMB-15 were used to design CMB-17S. Furthermore, it was found that the results of the developed collision model were within the criteria such as hourglass energy, mass change, negative volumes, Theoretical Head Impact Velocity (THIV), and Post-impact Head Deceleration (PHD) presented in Ray et al. [15] as well as CEN TR 16303 [16,17,18,19].

### 2.2. Deformable Concrete Median Barrier

In order to develop a new CMB under harsher conditions, materials such as fiber reinforced concrete (FRC) can be considered as good materials against impact or blast. Some studies on impact resistance of FRC have shown that FRC limits the extent of damage more than the ordinary concrete median barriers [20,21]. However, at the same time, the FRC can increase the construction cost. According to Iqbal et al. [22,23], the concrete structures with FRC also showed an increased impact resistance; however, the construction cost could be a big obstacle for installing CMB in long highway sections.

**Figure 2:**
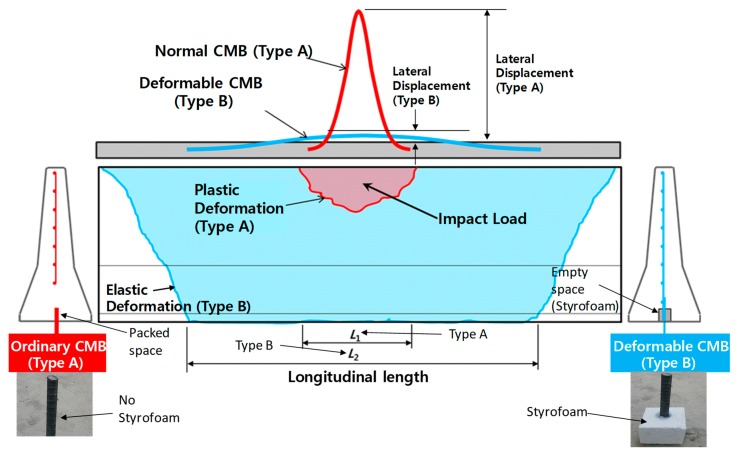
A conceptual drawing for comparison of an ordinary CMB (Type A) with a deformable CMB (Type B).

Accordingly, in this study, instead of using FRC for new design, the concept of deformable CMB was applied to the new design of CMB. The deformable concrete median barrier was first introduced by Kim et al. [2]. The small empty spaces were intentionally formed using Styrofoam at every location of the dowel bars so that the dowel bar could deform via shearing under impact loading. The deformed dowel bar due to the addition of Styrofoam under lateral load would then successfully absorb a significant portion of energy by allowing a certain displacement between the CMB and the foundation. Figure 2 shows lateral and longitudinal lengths. The lateral displacement indicates an amount of deformation that is normal to longitudinal direction, whereas the longitudinal length is a partial length with deformation along the length of the deformable CMB due to the impact. As Figure 2 shows, the ordinary CMB (Type A) showed a localized deformation that had a high possibility to produce large concrete fragmentations. However, a deformable CMB (Type B) not only benefitted from shock absorbers for absorbing impact energy but also dispersed deformations along the length of the CMB that dispersed the impact energy on a much larger area of CMB. These effects would significantly reduce any large fragmentation through localized impact. However, the performance of the deformable CMB strongly depends on the size of the dowel bar. Therefore, the size of dowel bar is one of the key parameters for developing CMB-17S, and a parametric study of the dowel bar is presented in a later section.

**Figure 3:**
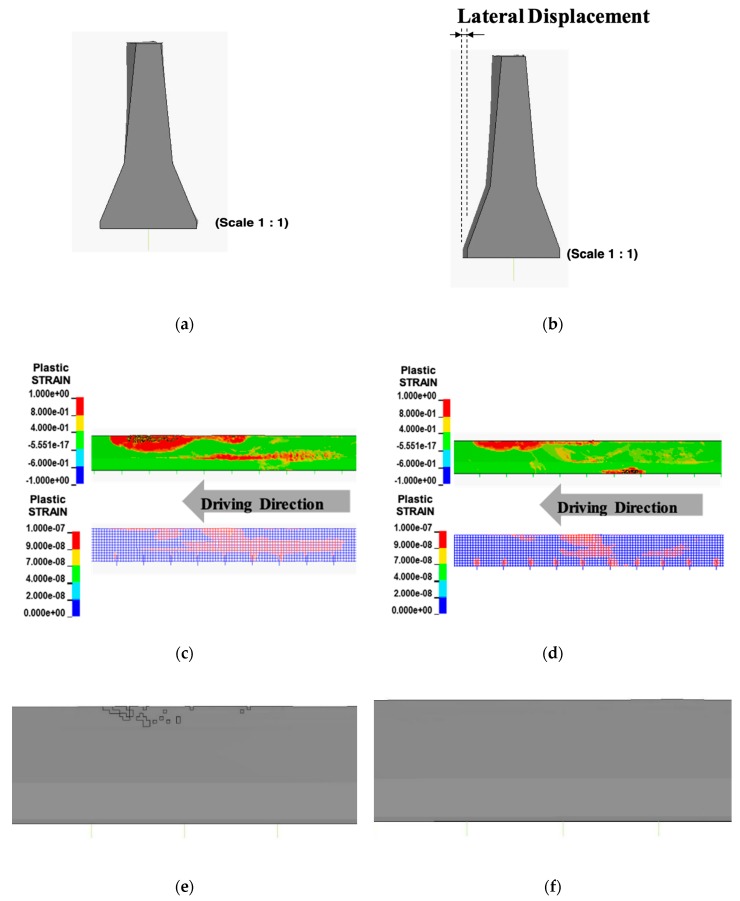
A comparison of deformable concrete median barriers (CMBs) (with shock absorbers) with an ordinary CMB (without shock absorbers). (**a**) Lateral displacement without shock absorbers; (**b**) lateral displacement with shock absorbers; (**c**) longitudinal length without shock absorbers; (**d**) longitudinal length with shock absorbers; (**e**) volume loss without shock absorbers; (**f**) volume loss with shock absorbers.

Figure 3 shows the effect of shock absorbers obtained from a simulation using the developed model. The lateral displacement was not observed from an ordinary CMB (Type A, Figure 3a) but was observed from a deformable CMB (Type B, Figure 3b) due to impact as intended. The zones in the plastic strain conditions of concrete and steel wire-mesh are shown in Figure 3c,d. The eroded elements could be only seen in an ordinary CMB (Type A, Figure 3e). A deformable CMB (Type B, Figure 3f) with shock absorbers showed negligible eroded elements. Large lateral displacement, longer longitudinal length, and less volume loss was obtained due to dispersed deformations along the length of the CMB that would disperse the impact energy on a much larger area of CMB, as previously explained. Similar effects due to the addition of shock absorbers can also be found in Kim et al. [2].

### 2.3. Parametric Study

For the section design of CMB-17S, a parametric study using the developed model was performed after verification of the model. A detailed discussion for a model verification can be found in Section 2.1. In a previous study conducted by Kim et al. [2,4], an additional 50 mm width of the CMB was applied while the slope of the side face was kept the same as that of the CMB-15. This new deformable CMB with shock absorbers was named as Hi-CMB [2] and designed for SB5-B(20A) (456 kJ). However, in this study, we call it CMB-16 since it was tested in 2016.

For developing CMB-17S in this study, the additional 50 mm width was not applied; instead, the same width and the same slope of the side face as those of the CMB-15 (named “2015 type” in Kim et al. [2]) were used as an overall shape of the new CMB. It was a main design philosophy in which, compared to the CMB-16, less concrete and reinforcement were used for new sections in order to effectively minimize the construction cost. However, at the same time, the new section should hold a similar deformable performance comparable to CMB-16 [2,4] under a severe impact level such as SB6. Therefore, only the reinforcement, which is wire-mesh, and the shock observers, which comprised void space filled with Styrofoam and dowel bars, were the key design parameters of CMB-17S.

**Figure 4:**
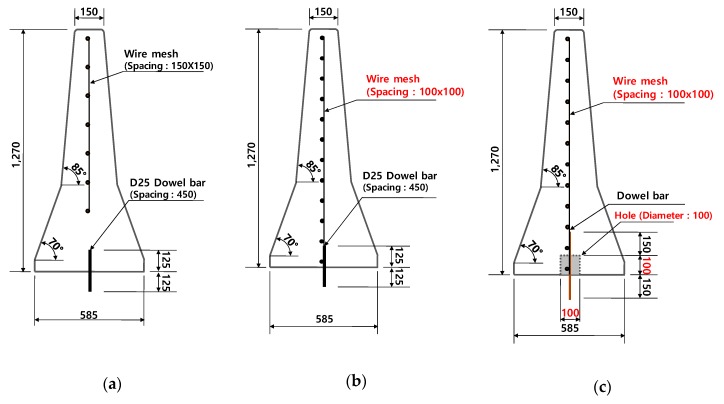
Current CMB (CMB-15) in service and the proposed Type A and Type B CMBs. (**a**) CMB-15; (**b**) fixed ordinary CMB (Type A); (**c**) deformable CMB with shock absorbers (Type B).

Accordingly, the following three designs—CMB-15 (currently used CMB in South Korea, Figure 4a), Type A (fixed without shock absorbers and strengthening reinforcement, Figure 4b), and Type B (deformable with shock absorbers and strengthening reinforcement, Figure 4c)—were compared with each other. Type A indicated a fixed boundary condition between the foundation (old concrete) and the bottom of the CMB (new concrete). Type B indicated that every design was the same except the addition of shock absorbers. Therefore, Type B was not any fixed type but a deformable CMB, which allowed lateral displacement to absorb the impact energy effectively. The details of the model parameters for shock absorbers using dowel bars, such as bond strength between the soffit of the CMB and the foundation and the height of the dowel bar can be found in Kim et al. [4]. Summarized simulation results for these three types of CMB are shown in Table 1.

#### 2.3.1. Obtained Results Depending on Size of Wire Mesh and Dowel Bar

**Figure 5:**
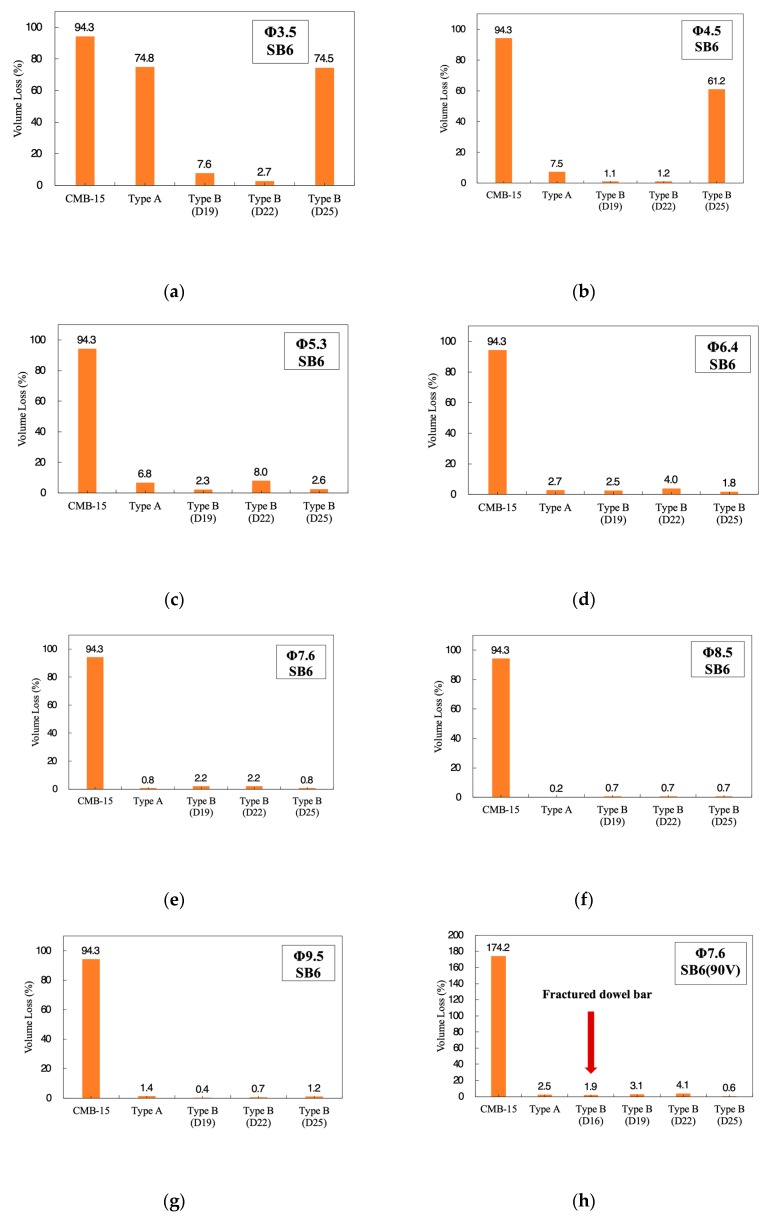
Obtained volume loss depending on the size of wire-mesh under SB6 impact severity. (**a**) Wire size—ø3.5; (**b**) Wire size—ø4.5; (**c**) Wire size—ø5.3 (SB6); (**d**) Wire size—ø6.4 (SB6); (**e**) Wire size—ø7.6 (SB6); (**f**) Wire size—ø8.5 (SB6); (**g**) Wire size—ø9.5 (SB6); (**h**) Wire size—ø7.6 (SB6(90V)).

The key parameters, namely, size (diameter) of wire mesh and dowel bar for shock absorbers, were considered in the parametric study, and the results are shown in Figure 5. Each graph shows results depending on the size of wire-mesh. The diameter of the wire-mesh ranged from 3.2 mm to 9.5 mm, while the spacing between the steel wires was fixed to 100 mm in both the longitudinal and the vertical directions. To reduce fragmentations under impact loading, smaller spacing was recommended for trapping concrete aggregates effectively. However, there should be a minimum limit of the spacing, because if too a small spacing is used, segregation due to the wire-mesh can occur. During the slip foam construction, short spacing between the wires (less than 75 mm) can split a single CMB into two based on experiences from construction specialists. Therefore, 100 mm wire-spacing was considered to be the minimum wire spacing for designing reinforcement in the body of CMB. The 100 mm spacing was also proven to be an optimal spacing from previous CMB-16 experiments [2]. No segregation due to existing wire-mesh was observed during construction. Therefore, the spacing was not a design parameter, and it was fixed to 100 mm in both the directions for this study. Three different dowel bars, namely, D19, D22, and D25, were considered, and the results were compared to each other. The spacing between the dowel bars of Type A and Type B were 300 mm and 1000 mm, respectively. The 1000 mm spacing for Type B dowel bar was already determined by Kim et al. [4].

In Figure 5a–g, the volume loss ratios (%) were compared. The volume loss ratio was calculated from the weight of the deleted concrete element from simulations or the weight of fragmentations from experiments due to impact divided by the total weight of a CMB of length 1.0 m. Acceptance criteria 3.5% was selected for the design of CMB-17S. The volume loss ratio, which is the weight of the total amount of fragmentations that occurred under the impact (SB5-B), was 7.0% for the CMB-15, and it finally obtained certification after passing all the test checklists. The 3.5% volume loss was 50% volume loss obtained from the field test of CMB-15. Therefore, the acceptable criteria for volume loss 3.5% was certainly a conservative design.

Both 3.5 and 4.5 mm wire-mesh (Figure 5a,b) showed volume loss ratios less than 3.5% for a few cases. It was interesting to note that 3.5 and 4.5 mm wire-mesh with D25 dowel bars (Type B) showed volume loss ratios equivalent or even more than the results of Type A CMB. It was thought that a larger size of dowel bar either delayed or did not trigger the effect of shock absorbers due to stiffer connection condition between the foundation and the bottom of the CMB compared to relatively smaller sizes such as D19 and D22. However, Type B CMB with D19 and D22 showed the effect of shock absorbers and volume loss ratio, which was less than Type A. This indicated that the wire-mesh of 3.5 mm with D22 dowel bar could be an alternative design for SB6 impact severity. However, for conservative design with unknown factors, such as severe damages due to the local impact that was observed from CMB-16, Type B CMB with wire-mesh size larger than 7.6 mm was considered; finally, 7.6 mm was selected as the wire-mesh size. Wire-mesh larger than or equal to 7.6 mm showed volume loss ratio less than 3.5% regardless of the type of dowel bar used. Therefore, 7.6 mm could be the optimal economical design for CMB-17S. Furthermore, under the harsher impact severity such as SB6(90V), it was found that the D16 dowel bar was not the option due to fracture (Figure 5h). Thus, more cases for the selection of the dowel bar were studied under the harsher impact condition. Accordingly, the harsher impact conditions such as SB6(100V) was considered to find a proper size for the dowel bars for both safer design and better maintenance of the deformable concrete barrier.

#### 2.3.2. Selection of Dowel Bar Size

**Figure 6:**
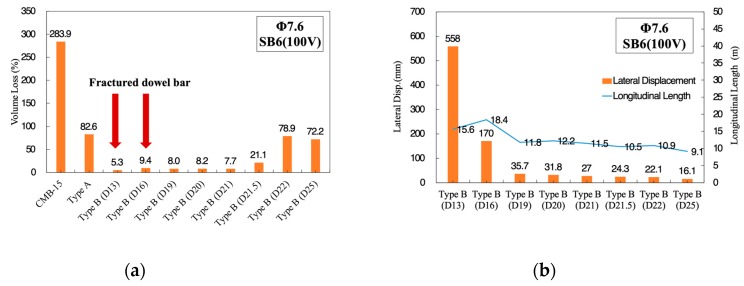
Obtained volume loss depending on the size of wire-mesh under SB6 impact severity. (**a**) Volume loss ratio of ø7.6 wire-mesh under SB6(100V); (**b**) lateral displacement and longitudinal length (ø7.6) under SB6(100V).

Type B deformable CMB was an efficient and conservative design compared to Type A, as shown previously. For selection of proper dowel bar size, a more severe level of impact severity SB6(100V) was considered, since the results from SB6 level showed similar results regardless of the size of dowel bar used when 7.6 mm or larger sized wire-mesh was used. SB6(100V) was the test level with a combination of 25 ton mass, 100 km/h impact speed, and 15° impact angle. It was named as SB6(100V), since only the impact speed (V) was changed, keeping the other conditions the same as those of SB6. Under the harsher impact severity of the SB6(100V) condition, D13 and D16 dowel bars were fractured, while the D19 resisted under SB6(100V), as shown in Figure 6a, and deformed with a proper lateral displacement, as shown in Figure 6b. Lateral displacement less than 40 mm was considered as a proper criterion by maintenance prospect. In general, at expressways in South Korea, the buffer zone between the side of CMB and the edge of the traffic lane has a 1.5 m gap.

**Figure 7:**
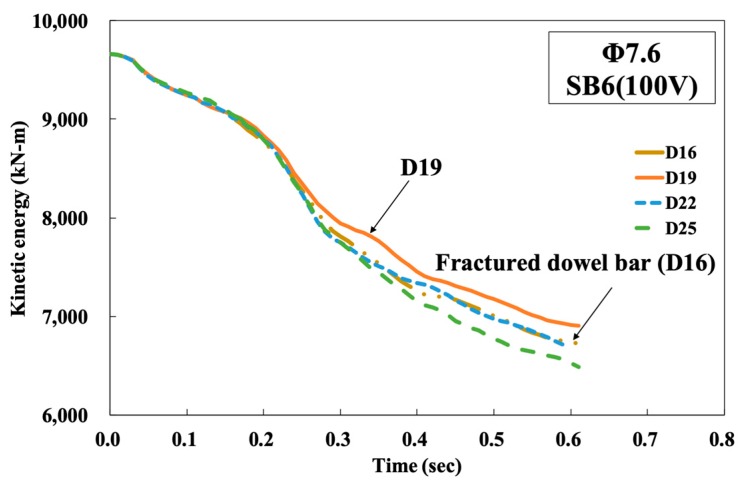
Kinetic energy of truck vs time under SB6(100V)).

The fractured dowel bar (D13 and D16 in Figure 6a) did not necessarily indicate poor performance, since it could absorb a larger amount of energy when both lateral displacement and longitudinal length occurred to large extents due to fracture. However, from the maintenance point of view, a dowel bar should not fracture, and large lateral displacement should also not occur. If kinetic energies of the trucks during a collision depending on the size of the dowel bar were compared among others, as shown in Figure 7, the largest kinetic energy of truck could be observed from D19. This indicated that the D19 dowel bar made the CMB absorb the smallest part of the truck’s kinetic energy, and the probability of generating concrete fragmentations was minimized under the SB6(100V) conditions. Accordingly, the D19 dowel bar was selected for the development of CMB-17S.

### 2.4. Proposal of CMB-17S

**Figure 8:**
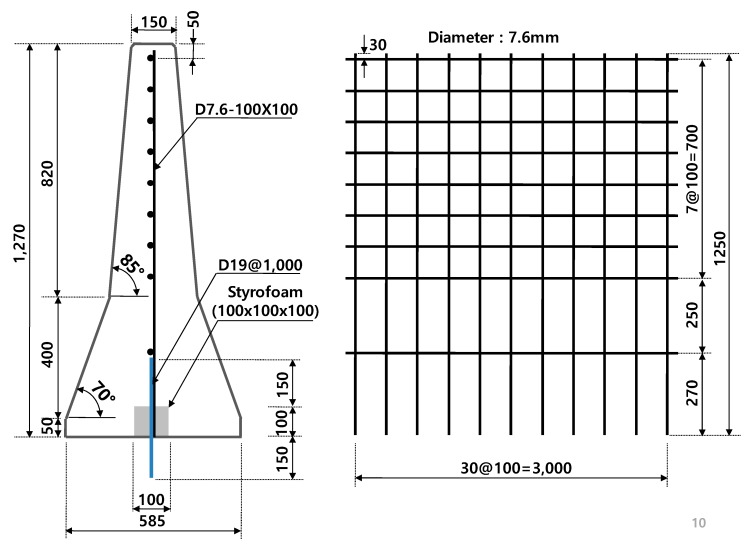
Proposed design of CMB-17S with a device allowing for deformation.

For selecting a proper dowel bar, two limits should be considered. One is the maximum limit of the size for designing a flexible dowel bar that can trigger the effect of the shock absorber, and another is the minimum limit of the size for designing a dowel bar from a maintenance point of view and in regard to the driver’s safety, who is driving in the opposite direction of the highway. A dowel bar larger than D19 showed somewhat stiff behavior, whereas a dowel bar smaller than D19 was fractured under severe impact load such as SB6(100V). Therefore, 7.6 mm wire-mesh with a D19 dowel bar was selected as the first design, and a field test was conducted for the final verification for low fragmentations, which were less than 3.5%. Furthermore, since most of the damage was localized at the upper part, it was not necessary to use the same spacing (100 mm) of wire-mesh in the lower part as that in the top part of the CMB. Accordingly, different spacings between the horizontal wires (which could reduce the reinforcement ratio in the lower part of CMB without losing any impact resistance capability) were attempted, and 250 to 270 mm spacing between the horizontal wires was selected for the lower part. The vertical wires were not removed, because the number of wires (steel ratio) in the top part would be reduced. The first design of CMB under SB6 was named as CMB-17S, as shown in Figure 8.

#### Comparisons of Damaged Zone between CMB-17S and CMB-15

**Figure 9:**
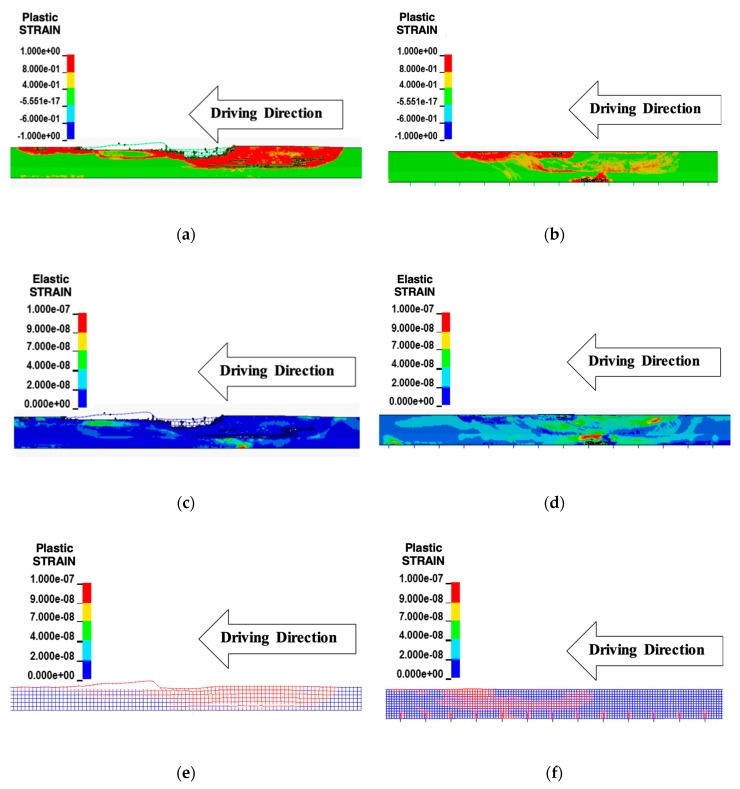
Damaged zones of CMB-15 and CMB-17S. (**a**) Plastic strain of concrete (CMB-15); (**b**) plastic strain of concrete (CMB-17S); (**c**) elastic strain of concrete (CMB-15); (**d**) elastic strain of concrete (CMB-17S); (**e**) plastic strain of wire-mesh (CMB-15); (**f**) plastic strain of wire-mesh (CMB-17S)**.**

Using a developed FE-model, the structural performance of CMB-17S was compared with that of CMB-15. In this study, elements in plastic strain conditions were considered as damaged zones. As shown in Figure 9a,b, the damaged zone and the eroded elements of the concrete were significantly reduced when a design of CMB-17S was compared to CMB-15. If the elastic strains from both designs were compared, the elastically strained region of CMB-17S was dispersed in a wider area than that of CMB-15, as shown in Figure 9c,d. This indicated that CMB-17S was deformed, and impact energy was widely distributed along the length of the CMB as intended. Figure 9e,f shows that the plastic strain of the wire-mesh in CMB-17S was diminished when compared to that of CMB-15. The number of elements in plastic strain conditions at the top part of the CMB decreased, while elements in plastic strain conditions around the dowel bar were newly generated from CMB-17S, as shown in Figure 9f. This indicated that dowel bars as shock absorbers were laterally deformed and absorbed a certain amount of impact energy, and the plastic strained zone at the top part of CMB-17S was reduced in size. Therefore, based on obtained results using the developed FE-model, the proposed design shown in Figure 8 was considered as an adequate design under the impact severity SB6.

## 3. Full-Scale Truck-CMB Collision Test

As previously explained, the new CMB-17S was proposed after conducting several numerical analyses. However, the obtained design was only associated with the simulation results. When a new design of concrete median barrier is developed for installation on a road, the new design becomes valid when full-scale truck-CMB collision test results meet the recommended performance criteria specified by the government [24]. In South Korea, a full-scale test has been the most common method for evaluating the safety performance of road safety features, such as median barriers and guardrails. Therefore, a full scale test of CMB-17S was performed and evaluated with respect to the performance criteria specified by the government [25].

### 3.1. Test Conditions and Criteria

**Figure 10:**
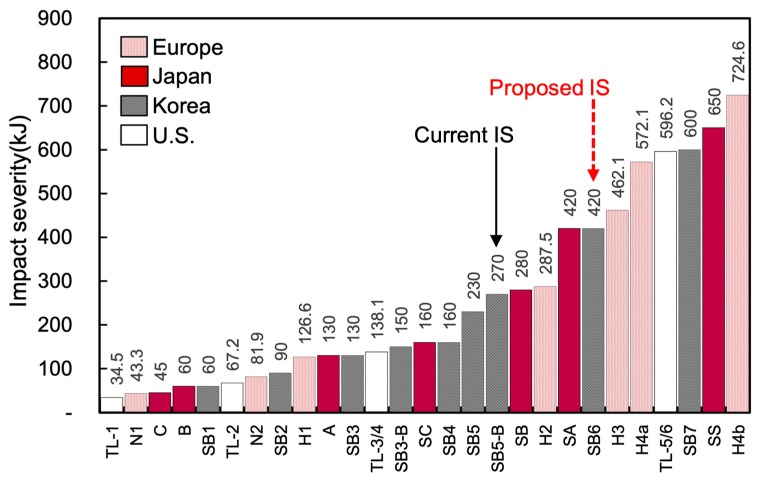
Comparison of impact severity (IS) among developed countries [25,26,27].

A comparison of the impact severity of different countries (Europe, Japan, Korea, and the U.S.) is presented in Figure 10. Most of the countries have already installed strong CMBs that have higher levels of impact severities, such as TL-5/6 in the United States. In South Korea, a high level of impact severity (SB7), which is equivalent to TL5/6, is also specified in the MOLIT [25] guideline. However, the maximum design level of the CMB in service in South Korea is still SB5-B, which has an impact energy of 270 kJ. Therefore, as previously explained, SB6(420 kJ) was used to develop the new CMBs. The specified impact conditions for SB6 and similar levels from other countries are compared in Table 2. According to MOLIT [25], 25 ton trucks were prepared, and full scale collision tests were performed.

### 3.2. Full Scale Collision Test between Truck and CMB

#### 3.2.1. Preparation of the Full-Scale Test

**Figure 11:**
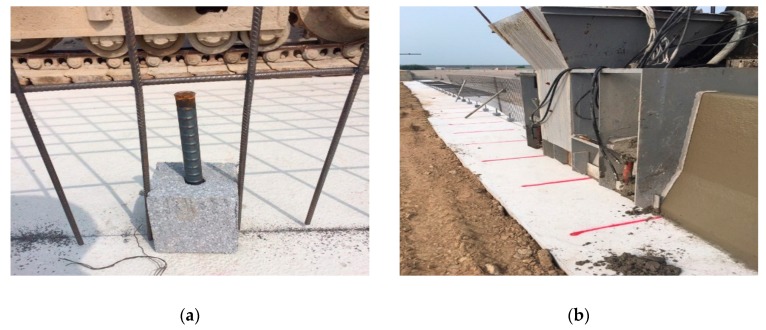
Installation of proposed CMB-17S. (**a**) Shock absorbing device (Dowel bar) for intended deformation; (**b**) Slip-forming concrete median barrier.

Before pouring concrete, the wire-meshes were aligned along the length of the CMB, and the D19 dowel bars were installed at a spacing of 1.0 m for an intended deformation, as shown in Figure 11a. The specified concrete compressive strength was 30 MPa, whereas the obtained strength when the test was conducted was 31.2 MPa. A slip form as shown in Figure 11b was used, and 30 m CMBs were constructed. After the final setting of the concrete, the CMBs were covered with several clothes, and water was sprinkled to keep the clothes wet in order to minimize any drying shrinkage during the summer season.

**Figure 12:**
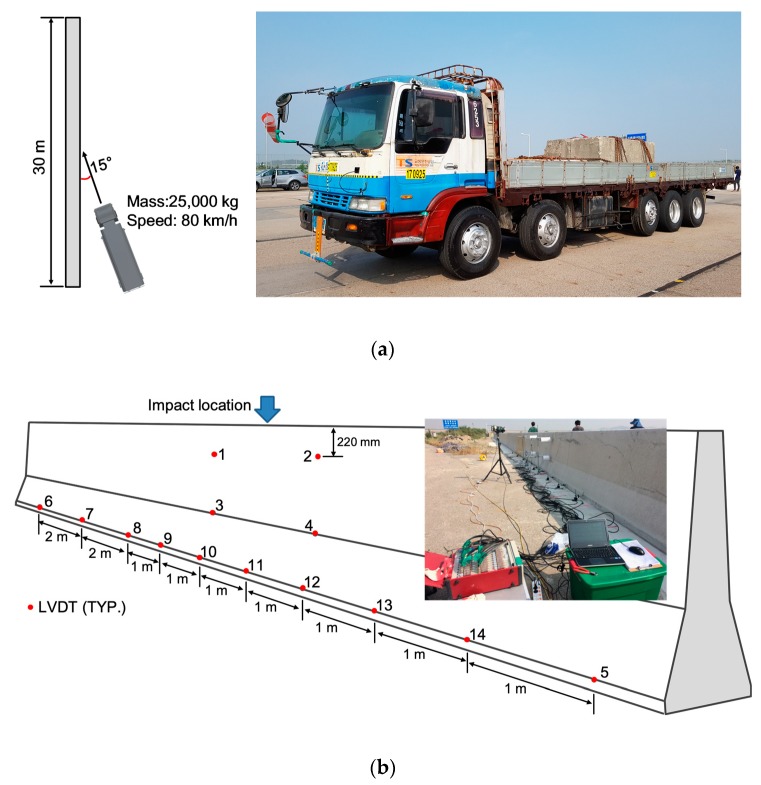
Test set-up. (**a**) Test vehicle; (**b**) Locations of the linear variable differential transformer (LVDT).

A 25 ton truck with a 14.5 ton curb weight was used, as shown in Figure 12a. Therefore, the concrete mass used in the cargo was 10.5 ton. According to SB6 criteria by MOLIT [24], the impact speed was 80 km/h, and the impact angle was 15°.

During the impact, the deformation of the CMB was carefully measured using several linear variable differential transformers (LVDTs), as shown in Figure 12b.

A total of 14 LVDTs were used to measure the lateral displacement of CMB-17S due to impacts. To evaluate the body deformation of CMB-17S, the four LVDTs (LVDT-1–LVDT-4 in Figure 12) were mounted on a steel frame, which was fixed to a concrete foundation slab. The other LVDTs (LVDT-5–LVDT-14 in Figure 12) were placed at 50 mm from the foundation slab at intervals of one meter or two meters to measure the deformed length of CMB-17S after the impact load (see Figure 12). The obtained results of the impact comprised 1000 Hz data points.

#### 3.2.2. Crash Test Results

**Figure 13:**
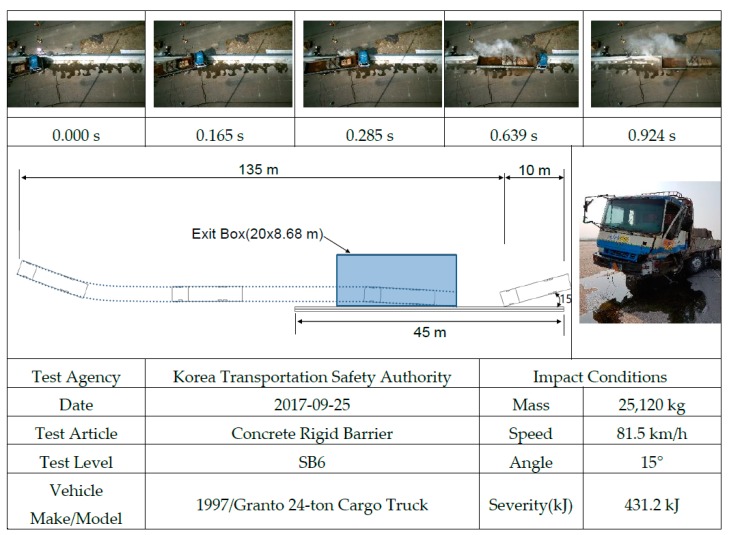
Summary of test results and sequential photographs.

A summary of the test results and the sequential photographs is shown in Figure 13. After a collision, the truck was satisfactorily induced in the designated lane and did not go beyond the exit box. To note, after a collision, if a truck goes beyond the exit box, then the CMB is considered to fail the test.

**Figure 14:**
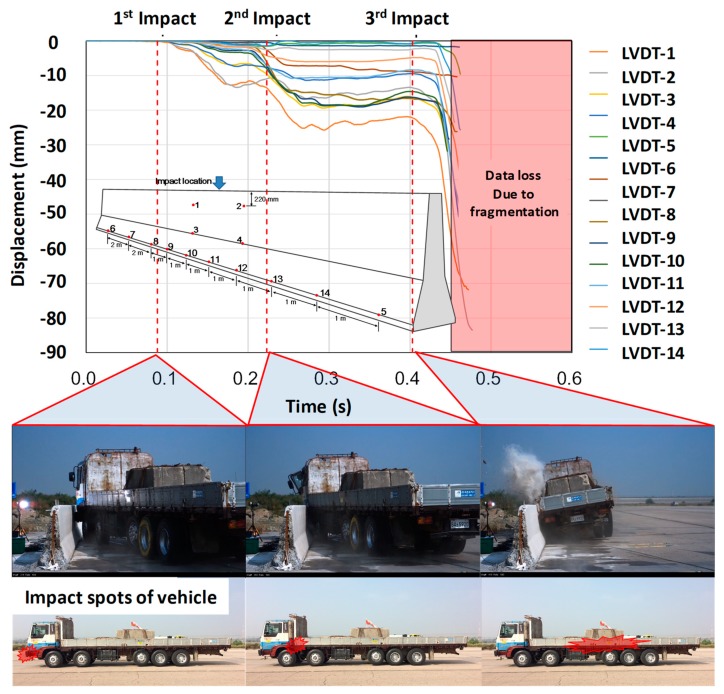
Obtained LVDTs’ data of concrete median barrier during impacts.

The obtained data from LVDTs are shown in Figure 14. During collision between the truck and CMB-17S, three major impacts were observed. The same impacts were also observed and discussed by Kim et al. [4].

First, the bumper zone was contacted with negligible concrete fragmentation. Second, the lower corner zone of the steel cargo compartment collided with the upper zone of the CMB, causing a large amount of concrete fragmentation and moderate lateral displacement. The third impact was a collision between the CMB and the truck rear wheels with negligible concrete fragmentation. However, during this impact stage, the maximum lateral displacement was observed, as shown in Figure 14.

**Figure 15:**
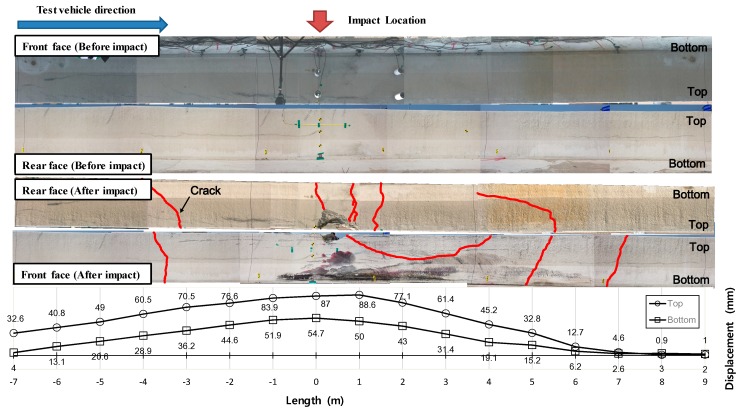
Lateral displacement along longitudinal direction of CMB-17S, measured with respect to several LVDTs.

All the deformed shape and crack patterns in front and rear faces after collision are illustrated in Figure 15. At the rear face, more vertical cracks were observed due to applied moment under impact loading. At the front face, U-shape cracks near the impact point were observed with some vertical cracks as well. The maximum deformation was observed at the top part of the impact point. The obtained permanent maximum deformation after elastic recovering behavior was 88.6 mm.

**Figure 16:**
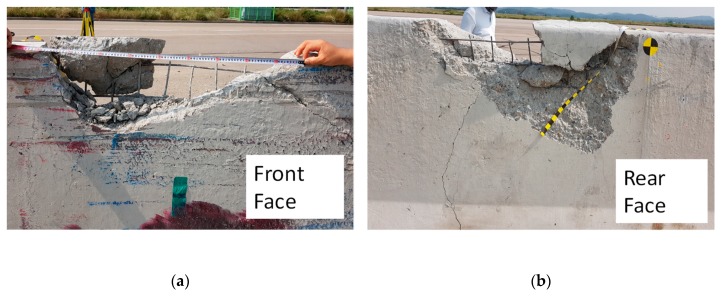

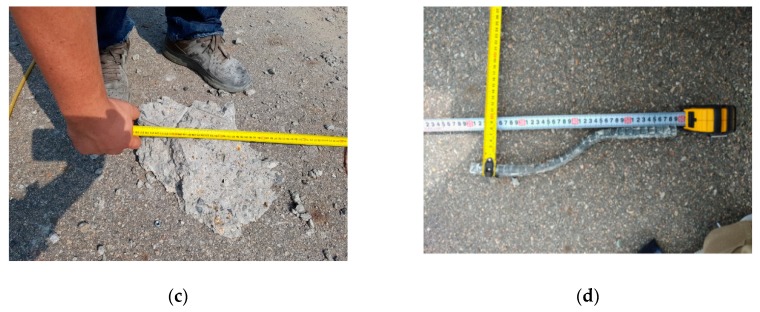
Photographs of CMB-17S after impacts. (**a**) Front face; (**b**) Rear face; (**c**) Concrete fragmentation 2.1 m away from CMB-17S; (**d**) Deformed dowel bar of the device allowing for deformation.

Figure 16 shows the concrete fragmentations caused by local collision between the lower corner zone of the steel cargo compartment and the upper zone of CMB-17S. Due to the local impact from the lower corner zone of the steel cargo compartment, stress concentration occurred, causing a large amount of concrete fragmentation, as shown in Figure 16a–c. This amount of fragmentation due to local impact could not be accurately predicted from the developed numerical model. The destroyed shape of the CMB-17S after local second impact could be considered as a punching shear failure.

A deformed dowel bar after impact was also observed, as shown in Figure 16d, which indicated that the installed dowel bar type shock absorbers worked as intended.

#### 3.2.3. Comparison of the Numerical Model with the Test Results

The obtained results from the full-scale field test and the numerical analysis were compared with each other. For numerical analysis, the truck model had the same total mass as that of the field test. However, the curb weights for the truck model and the real tested truck were 14 ton and 14.5 ton, respectively. The dimensions of the truck model were 2,486 mm width, 2,966 mm height, and 10,018 mm length, while the tested real truck had 2,490 mm width, 2,970 mm height, and 12,582 mm length. Moreover, the actual material properties of the truck parts could have been different from the input values in the numerical model for the materials of the truck parts.

As previously mentioned, the volume loss ratio was not well predicted by the developed model. Based on the results from the developed model, 2.2% volume loss was predicted. However, due to local impact (second impact), a larger amount of volume loss (8.7%) was measured. This indicated that the developed model was not accurate for estimating volume loss caused by local impact (second impact). Furthermore, it was found that a larger amount of steel ratio was required for the top part of the CMB under SB6 load.

**Figure 17:**
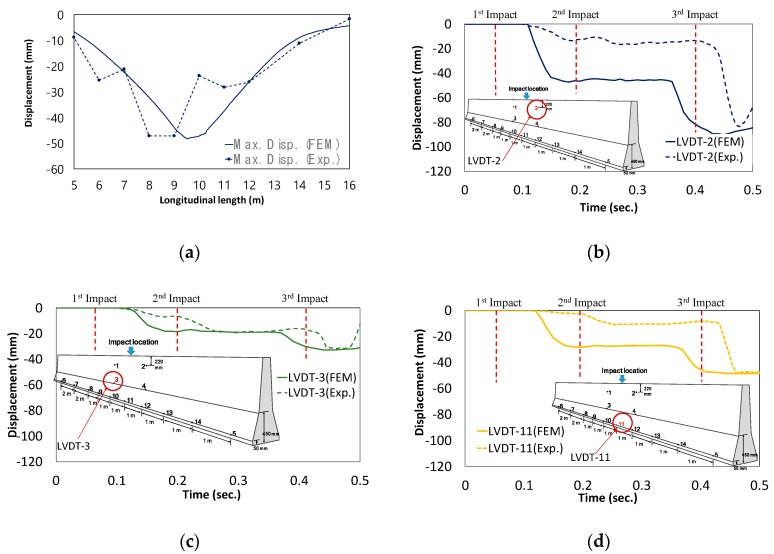
Comparison between numerical and experimental results. (**a**) Deformed shape; (**b**) LVDT (#2); (**c**) LVDT (#3) data from bottom right part; (**d**) LVDT (#11) data from bottom left part.

The maximum deformations obtained along the length of CMB-17S from both testing and analysis had similar results, as shown in Figure 17a. The maximum deformation was during impact loading before any elastic recovery of the deformed CMB. The LVDTs at location 12 had a 48.14 mm deformation maximum at the bottom of CMB-17S, while numerical analysis predicted 47.23 mm (a difference of 1.9%). The locations of the maximum deformation from the field test and the analysis were 1.6 m apart (as shown in Figure 17a), when the total length of CMB-17S was 30 m.

The phase difference between the test and the analysis during the impact sequence is shown in Figure 17b–d. In the analysis, a large amount of deformation was observed during the first impact. There was negligible deformation during the second impact, and the largest deformation was observed during the third impact. However, in the field test, negligible deformation occurred during the first impact. This indicated that the test truck had a better bumper for energy absorption than the model, minimizing the first impact loading in actual test conditions and maximizing the effects of both the second and the third impacts. It is thought that the phase difference was caused by having different geometries and materials between the simulation model and the actual truck tested.

## 4. Conclusions

A new concrete median barrier (CMB-17S) was designed under impact severity SB6(420 kJ). While the current design (SB5-B, 270 kJ) can cover 50% of the total vehicle-CMB collision accidents, the newly proposed design level (SB6, 420 kJ) could cover 85% of the total vehicle-CMB collision accidents in South Korea. In the present study, no FRC was considered, nor was the cross-sectional shape of CMB changed. Keeping all the other conditions the same as in CMB-15, only the designs of the wire-mesh and the dowel bar type shock absorbers were modified. Several past parametric studies have adopted various dowel bars and wire-meshes to find the optimal design under SB6. The proposed new design in the current study, CMB-17S, was finally tested in the field with a full-scale truck. As intended, the lateral displacement of the shock absorbers occurred between the soffit of the CMB and the surface of the concrete foundation. However, a relatively larger amount of concrete fragmentation was observed from local impact by the tested truck. The following conclusions can be drawn from the current study.

1. In this study, a deformable concrete median barrier was designed and tested. The small empty spaces were intentionally formed using the Styrofoam at every location of dowel bars so that the dowel bar could deform via shearing under impact loading.
2. A deformable CMB could experience shock absorbing effects as well as dispersed deformations along the length of the CMB, which dispersed the impact energy over a much larger area of CMB.
3. A numerical model was developed to find volume loss under truck impact. In the model, a deformed dowel bar was modeled to predict any lateral displacement. After verification of the model, several parametric studies were conducted. The selected parameters were dowel bar size and wire-mesh size.
4. From the numerical analysis, it was found that the deformed dowel bar due to addition of Styrofoam under lateral load could successfully absorb a significant portion of energy by allowing a certain displacement between the CMB and the foundation.
5. The final proposed design comprised one layer of 7.6 mm wire-mesh and D19 dowel bars. The spacing between the wires was fixed to 100 mm, while the spacing between the dowel bars was fixed to 1000 mm.
6. It was found that the dowel bars that had a relatively smaller size (D13 or D16) were fractured under a harsher loading condition such as SB6(100V). By contrast, the larger dowel bars, such as D22 or D25, did not deform or absorb the energy fully and resulted in a larger amount of concrete fragmentation.
7. A full-scale collision test between a 25 ton truck and the developed CMB-17S was performed, and the installed dowel bar worked fine as intended.
8. Due to local impact from the lower corner zone of the steel cargo compartment at second impact, stress concentration occurred, causing a large amount of concrete fragmentation. This phenomenon was not accurately captured by our developed model. For more accurate prediction of volume loss, development of a local model is required.
9. A comparison of the analysis and the test results revealed that the final permanent deformation could be well captured by the developed model; however, volume loss, location of the maximum deformation, and sequence of the three major impacts were rather different. It is thought that this difference was due to the differences between the actual dimension and the material properties of the truck and the truck model.
10. For better performance of our developed CMB-17S, the top part of the CMB should be better strengthened by more wire-mesh, since major volume loss occurred due to local impact, which appeared to be due to punching shear failure.

## Figures and Tables

**Table 1:** The obtained results depending on types of CMBs.

CMB Type	Wire-Mesh Size (mm)	Wire-Mesh Spacing (mm)	Dowel Bar Size (mm)	Volume Loss Ratio (%)	Lateral Disp. (mm)	Impact Severity
CMB-15	3.2	150 × 150	25	94.3	N/A	SB6
Type A	3.5	100 × 100	25	74.8	N/A	SB6
	4.5	100 × 100	25	7.5	N/A	SB6
	5.3	100 × 100	25	6.8	N/A	SB6
	6.4	100 × 100	25	2.7	N/A	SB6
	7.6	100 × 100	25	0.8	N/A	SB6
	8.5	100 × 100	25	0.2	N/A	SB6
	9.5	100 × 100	25	1.4	N/A	SB6
Type B	3.5	100 × 100	19	7.6	29.1	SB6
	3.5	100 × 100	22	2.7	18.5	SB6
	3.5	100 × 100	25	74.5	12.2	SB6
	4.5	100 × 100	19	1.1	28	SB6
	4.5	100 × 100	22	1.2	18	SB6
	4.5	100 × 100	25	61.2	12.6	SB6
	5.3	100 × 100	19	2.3	29.7	SB6
	5.3	100 × 100	22	8.0	24.7	SB6
	5.3	100 × 100	25	2.6	12.5	SB6
	6.4	100 × 100	19	2.5	30.1	SB6
	6.4	100 × 100	22	4.0	19.6	SB6
	6.4	100 × 100	25	1.8	15.2	SB6
	7.6	100 × 100	19	2.2	31.1	SB6
	7.6	100 × 100	22	2.2	20.6	SB6
	7.6	100 × 100	25	0.8	15.9	SB6
	7.6	100 × 100	13	5.3	558	SB6(100V)
	7.6	100 × 100	16	9.4	170	SB6(100V)
	7.6	100 × 100	19	8.0	35.7	SB6(100V)
	7.6	100 × 100	20	8.2	31.8	SB6(100V)
	7.6	100 × 100	21	7.7	27	SB6(100V)
	7.6	100 × 100	21.5	21.1	24.3	SB6(100V)
	7.6	100 × 100	22	78.9	22.1	SB6(100V)
	7.6	100 × 100	25	72.2	16.1	SB6(100V)
	8.5	100 × 100	19	0.7	29.2	SB6
	8.5	100 × 100	22	0.7	20.5	SB6
	8.5	100 × 100	25	0.7	15.7	SB6
	9.5	100 × 100	19	0.4	29	SB6
	9.5	100 × 100	22	0.7	21.1	SB6
	9.5	100 × 100	25	1.2	14.5	SB6

**Table 2:** Comparison of impact test conditions among developed countries [25,26,27,28].

Impact Conditions	Korea (MOLIT 2015)		Japan (JRA 2008)	Europe (CEN 1998)	U.S. (AASHTO 2016)
	SB5-B	SB6	SA	H3	TL6
Mass (kg)	14,000	25,000	25,000	16,000	36,000
Impact speed (km/h)	85	80	80	80	80
Impact angle (°)	15	15	15	20	15
Impact severity (kJ)	270	420	420	462.1	600
